# The chemical structure and phosphorothioate content of hydrophobically modified siRNAs impact extrahepatic distribution and efficacy

**DOI:** 10.1093/nar/gkaa595

**Published:** 2020-07-16

**Authors:** Annabelle Biscans, Jillian Caiazzi, Sarah Davis, Nicholas McHugh, Jacquelyn Sousa, Anastasia Khvorova

**Affiliations:** RNA Therapeutics Institute, University of Massachusetts Medical School, Worcester, MA 01604, USA; Program in Molecular Medicine, University of Massachusetts Medical School, Worcester, MA 01604, USA; RNA Therapeutics Institute, University of Massachusetts Medical School, Worcester, MA 01604, USA; Program in Molecular Medicine, University of Massachusetts Medical School, Worcester, MA 01604, USA; RNA Therapeutics Institute, University of Massachusetts Medical School, Worcester, MA 01604, USA; Program in Molecular Medicine, University of Massachusetts Medical School, Worcester, MA 01604, USA; RNA Therapeutics Institute, University of Massachusetts Medical School, Worcester, MA 01604, USA; Program in Molecular Medicine, University of Massachusetts Medical School, Worcester, MA 01604, USA; RNA Therapeutics Institute, University of Massachusetts Medical School, Worcester, MA 01604, USA; Program in Molecular Medicine, University of Massachusetts Medical School, Worcester, MA 01604, USA; RNA Therapeutics Institute, University of Massachusetts Medical School, Worcester, MA 01604, USA; Program in Molecular Medicine, University of Massachusetts Medical School, Worcester, MA 01604, USA

## Abstract

Small interfering RNAs (siRNAs) have revolutionized the treatment of liver diseases. However, robust siRNA delivery to other tissues represents a major technological need. Conjugating lipids (e.g. docosanoic acid, DCA) to siRNA supports extrahepatic delivery, but tissue accumulation and gene silencing efficacy are lower than that achieved in liver by clinical-stage compounds. The chemical structure of conjugated siRNA may significantly impact *invivo* efficacy, particularly in tissues with lower compound accumulation. Here, we report the first systematic evaluation of the impact of siRNA scaffold—i.e. structure, phosphorothioate (PS) content, linker composition—on DCA-conjugated siRNA delivery and efficacy *in vivo*. We found that structural asymmetry (e.g. 5- or 2-nt overhang) has no impact on accumulation, but is a principal factor for enhancing activity in extrahepatic tissues. Similarly, linker chemistry (cleavable versus stable) altered activity, but not accumulation. In contrast, increasing PS content enhanced accumulation of asymmetric compounds, but negatively impacted efficacy. Our findings suggest that siRNA tissue accumulation does not fully define efficacy, and that the impact of siRNA chemical structure on activity is driven by intracellular re-distribution and endosomal escape. Fine-tuning siRNA chemical structure for optimal extrahepatic efficacy is a critical next step for the progression of therapeutic RNAi applications beyond liver.

## INTRODUCTION

Small interfering RNA (siRNA)-based therapeutics are revolutionizing human medicine, particularly for liver indications. Indeed, chemically modified N-acetylgalactosamine (GalNAc)-conjugated siRNAs demonstrate robust efficacy in liver (1–3), with a single subcutaneous injection supporting a duration of effect of up to 12 months without adverse events (4–6). However, as of today, clinically efficient siRNAs are limited to liver only. To expand therapeutic siRNA delivery to tissues beyond liver, lipid conjugation strategies have been explored (7–12).

Recently, we found that docosanoic acid (DCA)-conjugated siRNAs support functional delivery to a wide range of tissues, including muscle, heart, fat, adrenal glands and lung, without causing overt toxicity (13,14). Importantly, DCA enabled 3- to 9-fold higher siRNA levels in extrahepatic tissues compared to cholesterol, a more widely used hydrophobic conjugate (13). However, the level of extrahepatic silencing (30–60%) remains significantly lower than that routinely achieved in the liver (80–90%). Thus, further optimization of conjugated siRNAs targeting extrahepatic tissues is needed.

For conjugate-mediated delivery, full chemical stabilization of siRNAs is required (15–17) to prevent rapid degradation during tissue distribution and intracellular relocalization and to enable long duration of effect. In general, chemical scaffolds that replace every 2′ hydroxyl (18–20), modify terminal nucleotide linkages (21,22) and stabilize the 5′ phosphate (23–26) are able to enhance stability, minimize innate immunity and maximize *in vivo* activity of conjugated siRNAs. However, the nature and position of the chemical modifications within siRNA may significantly impact their degree and duration of effect (17,27). For example, the extent of phosphorothioate (PS) modifications, which have been shown to impact protein binding (28–31), can significantly alter both *in vitro* and *in vivo* siRNA cellular uptake, trafficking and accumulation (21,30). Additionally, siRNA structure—e.g. asymmetric siRNAs containing a single-stranded overhang—may alter efficacy by enhancing passive cellular uptake and improving recognition by the RNA-induced silencing complex (RISC) (15,32–35). Finally, the chemical linker connecting the conjugate to siRNA may influence compound efficacy *in vivo*, which was observed with antisense oligonucleotides (ASO) (36). Thus, fine-tuning the chemical and structural nature of conjugated siRNAs may provide an opportunity to enhance gene silencing in tissues beyond liver. However, the effect of chemical structure on siRNA distribution and efficacy in extrahepatic tissues has never been systematically evaluated; and thus, is not fully understood.

**Figure 1.**
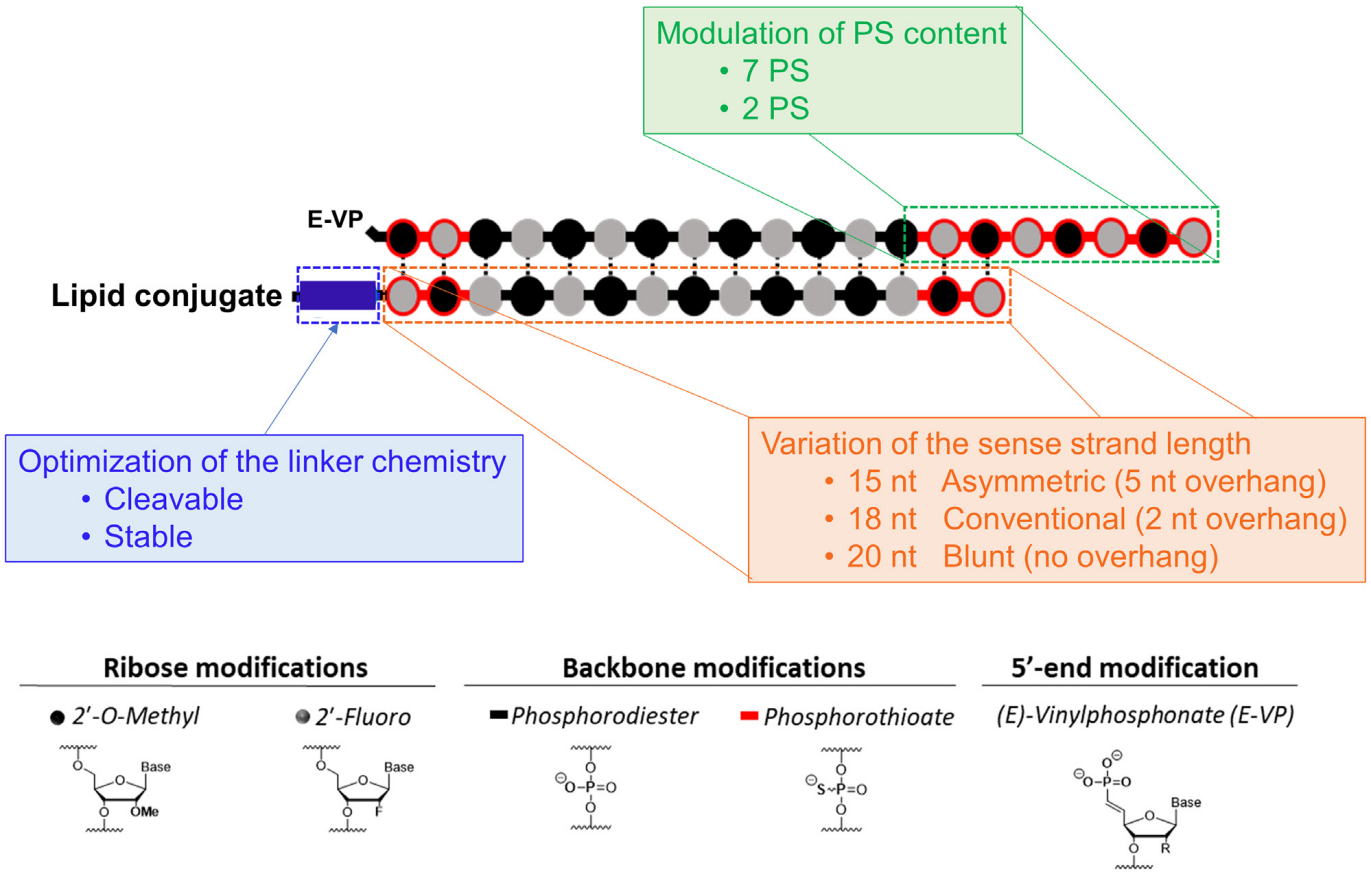
Variation of siRNA chemical structure, PS content and linker chemistry to evaluate the impact of these three major features on tissue distribution and efficacy *in vivo*.

Here, we methodically evaluated how altering chemical structure, PS content and linker chemistry of conjugated siRNAs affects extrahepatic tissue distribution and activity *in vivo* (Figure 1). To this end, we synthesized and delivered a panel of diverse DCA-conjugated siRNAs with asymmetric (5-nucleotide (nt) overhang), conventional (2-nt overhang) or blunt (no overhang) ends, varying numbers of PS modifications, and differing linker chemistries to mice. We found that, even with similar tissue accumulation, asymmetric and conventional siRNAs induce better silencing than blunt siRNAs in kidneys, spleen, heart, lung, muscle and adrenal glands. Using a cleavable linker also enhanced silencing activity across tissues. Conversely, increasing PS content was detrimental for functional efficacy in tissues despite being essential for siRNA stability and enhancing accumulation. Our findings demonstrate that improved extrahepatic efficacy of siRNAs requires combinatorial optimization of conjugate identity, siRNA chemical structure, PS content and linker chemistry.

## MATERIALS AND METHODS

### Oligonucleotide synthesis

A MerMade 12 synthesizer was used to synthesize oligonucleotides following standard protocols. DCA-conjugated sense strands were synthesized at 10 μmole scales on custom synthesized DCA-functionalized controlled pore glass (CPG) supports (13). Antisense strands were synthesized at 10 μmole scales on CPG functionalized with Unylinker^®^ (ChemGenes, Wilmington, MA, USA). All phosphoramidites were prepared as 0.15 M solutions in dry acetonitrile, and coupled using 0.25 M 5-(benzylthio)-1H-tetrazole (BTT) in acetonitrile as an activator for 250 s. Trityl groups were removed using 3% dichloroacetic acid in dichloromethane for 80 s. Unreacted 5′ hydroxyls on the growing oligonucleotide chain were capped with 16% N-methylimidazole in tetrahydrofuran (CAP B) and 80:10:10 (v/v/v) tetrahydrofuran:acetic anhydride:2,6-lutidine (CAP A) for 15 s. Sulfurizations were carried out with 0.1 M solution of 3-[(dimethylaminomethylene)amino]-3H-1,2,4-dithiazole-5-thione (DDTT) in acetonitrile for 3 min. Oxidation was performed with 0.02 M iodine in tetrahydrofuran:pyridine:water (70:20:10, v/v/v) for 80 s.

### Deprotection and purification of oligonucleotides

Sense strands were cleaved and deprotected using 40% aq. methylamine at 45°C for 1 h or ammonia-methylamine (AMA) at room temperature for 2 h. Antisense strands were first deprotected with a solution of bromotrimethylsilane:pyridine (3:2, v/v) in dichloromethane (5 ml) for (E)-vinylphosphonate deprotection, then cleaved and deprotected with 40% aq. methylamine at 45°C for 1 h or AMA at room temperature for 2 h. After drying overnight under vacuum (Speedvac), the resulting oligonucleotide pellets were suspended in water and purified using an Agilent Prostar System (Agilent, Santa Clara, CA, USA). Sense strands were purified over a Hamilton HxSil C18 column in a continuous gradient of sodium acetate: 90% Buffer A1 (50 mM sodium acetate in 5% acetonitrile) and 10% Buffer B1 (acetonitrile) to 10% Buffer A1 and 90% Buffer B1 at a flow rate of 30 ml/min for 18 min at 60°C. Antisense strands were purified over an ion-exchange column (GE Source 15Q media) in a continuous gradient of sodium perchlorate: 100% Buffer A2 (10 mM sodium acetate in 20% acetonitrile) to 60% Buffer A2 and 40% Buffer B2 (1 M sodium perchlorate in 20% acetonitrile) at a flow rate of 30 ml/min for 30 min at 60°C. Purified oligonucleotides were desalted by size-exclusion chromatography and characterized by Liquid Chromatography-Mass Spectrometry (LC-MS) analysis on an Agilent 6530 accurate-mass Q-TOF LC/MS (Agilent technologies, Santa Clara, CA, USA).

### Injection of conjugated siRNAs into mice

Animal experiments were performed in accordance with animal care ethics approval and guidelines of the University of Massachusetts Medical School Institutional Animal Care and Use Committee (IACUC, protocol number A-2411). Six- to seven-week-old female FVB/NJ mice (*n* = 5 per group) were injected subcutaneously with DCA-conjugated siRNA (20 mg/kg), a non-targeting control (Ntc) siRNA (20 mg/kg) or phosphate-buffered saline (PBS).

### Peptide nucleic acid (PNA) hybridization assay

At 1-week post-injection, the amount of siRNA antisense strand in tissues was determined using a peptide nucleic acid (PNA) hybridization assay, as described (37,38). Briefly, tissues (15 mg) were lysed in 300 μl MasterPure tissue lysis solution (EpiCentre) containing 0.2 mg/ml proteinase K (Invitrogen). Sodium dodecyl sulphate was precipitated from lysates by adding 20 μl 3 M potassium chloride, and pelleted by centrifugation at 5000 × *g* for 15 min. DCA-conjugated siRNAs in cleared supernatant were hybridized to a Cy3-labeled PNA probe fully complementary to the antisense strand (PNABio, Thousand Oaks, CA, USA). Samples were analyzed by HPLC (Agilent, Santa Clara, CA, USA) over a DNAPac PA100 anion-exchange column (Thermo Fisher Scientific). Cy3 fluorescence was monitored and peaks integrated. Final concentrations were ascertained using calibration curves.

### *In vivo* mRNA silencing experiments

At 1-week post-injection, tissues were collected and stored in RNAlater (Sigma) at 4°C overnight. mRNA was then quantified using the QuantiGene 2.0 Assay (Affymetrix). Briefly, tissue punches were lysed in 300 μl Homogenizing Buffer (Affymetrix) containing 0.2 mg/ml proteinase K (Invitrogen). Diluted lysates and probe sets (mouse *Htt*, mouse *Ppib*, or mouse *Hprt*) were added to the bDNA capture plate and signal was amplified and detected as described by Coles *et al.* (39). Luminescence was detected on a Tecan M1000 (Tecan, Morrisville, NC, USA).

### Statistical analysis

Data were analyzed using GraphPad Prism 8.1.2 software (GraphPad Software, Inc., San Diego, CA, USA). For each independent experiment in mice, the level of silencing was normalized to the mean of the PBS control group. Data were analyzed using non-parametric one-way ANOVA with Dunnett's test for multiple comparisons, and significance was calculated relative to PBS controls. *T*-tests were used for comparison between two groups.

## RESULTS

### The presence of a PS-modified 2- or 5-nt overhang does not alter DCA-siRNA tissue accumulation, but does enhance extrahepatic efficacy

#### Design of siRNA chemical structures

A wide variety of siRNA structures, including asymmetric siRNAs with five-seven nucleobase overhangs (15,33–35), conventional siRNAs with two-nucleobase overhangs (17,40), blunt compounds (41) and Dicer substrates (42,43), have been shown to be active *in vitro* and *in vivo*. However, the effect of these structures on the extrahepatic distribution and efficacy of siRNAs has not been systematically determined.

**Figure 2.**
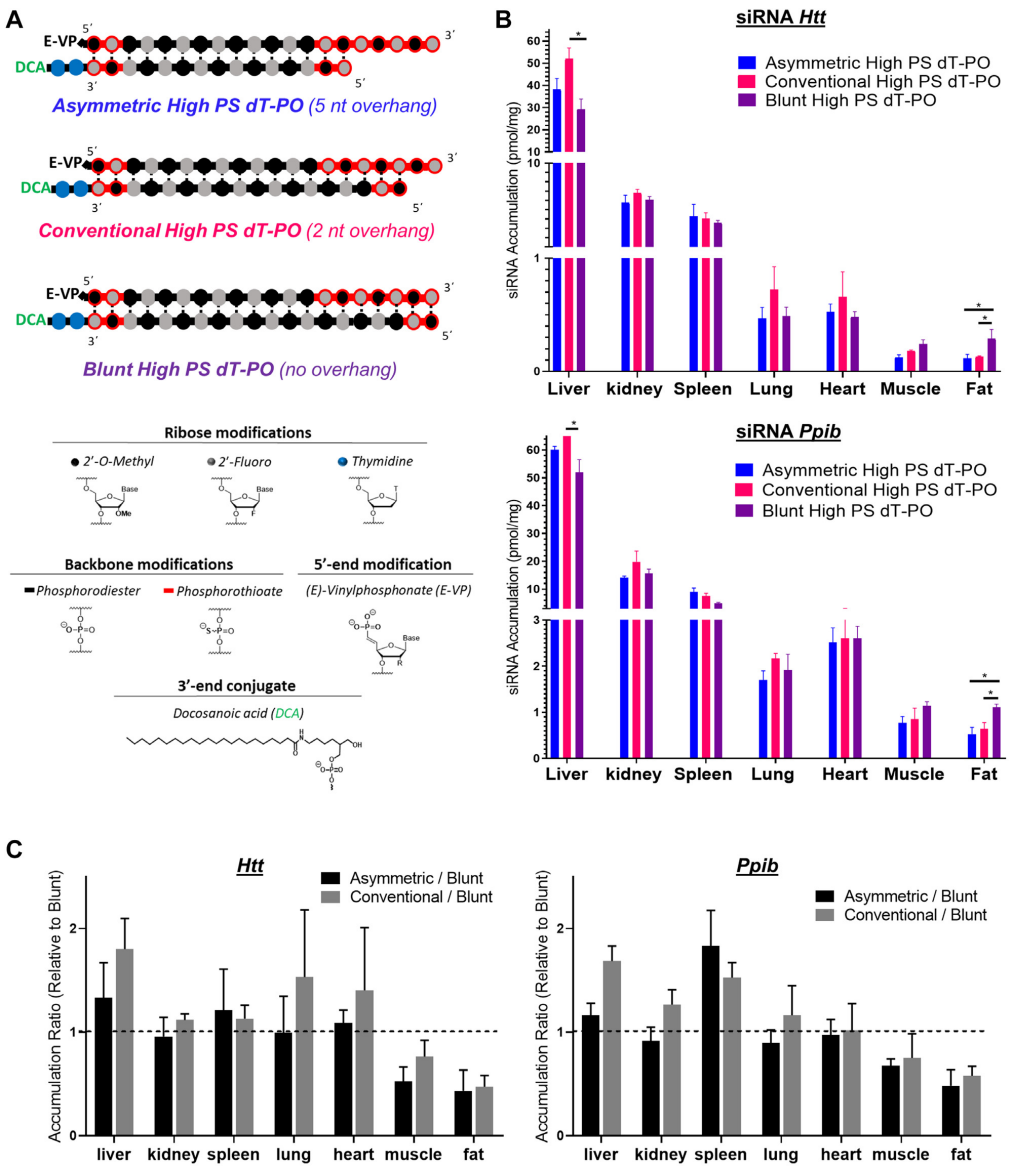
siRNA structure and presence of the PS-modified overhang does not impact tissue distribution and accumulation profiles. (**A**) Schematic of siRNA chemical structures used to evaluate the impact of phosphorothioate overhang length on siRNA extrahepatic distribution and efficacy. (**B**) Bar graph showing accumulation of DCA-conjugated siRNA targeting *Htt* (top) or *Ppib* (bottom) mRNA in liver, kidney, spleen, lung, heart, muscle and fat. siRNA accumulation measured 1-week after a single subcutaneous injection of DCA-siRNAs (20 mg/kg; *n* = 5–6 mice per group ± SD) by PNA hybridization assay. Data analysis: *t*-test (*P< 0.05). (**C**) Bar graph showing the tissue accumulation ratio of asymmetric or conventional siRNAs to blunt siRNA targeting *Htt* (left) or *Ppib* (right) mRNA.

To evaluate the effect of siRNA structure on distribution and efficacy, we selected three different siRNA scaffolds (Figure 2A). For all compounds, an alternating 2′-*O*-methyl and 2′-fluoro modification pattern with terminal PS linkage stabilization was used (18,21–22). Moreover, the antisense strand was modified with a 5′-(*E*)-vinylphosphonate (E-VP) group that mimics the 5′-phosphate of the antisense strand to promote recognition by RISC (23,24) and provide stability against phosphatases and exonucleases (25,26). To support broad distribution and silencing in a wide range of tissues—i.e. liver, heart, lung, fat, muscle, adrenal gland and spleen (13,14)—a docosanoic acid (DCA) conjugate was attached at the 3′-end of the sense strand through two phosphodiester bonds between two thymidines (dT-PO linker) (7,44–45). Finally, all three siRNA variants had the same 20-base antisense strand chemical structure containing 9 PS modifications.

The only variation in the siRNA scaffold was the length of the sense strand, which dictated the length of the single-stranded PS overhang. Specifically, we used a 15-base sense strand to generate a compound with a 5-nucleotide (nt) PS overhang (asymmetric siRNA); an 18-base sense strand to generate a 2-nt PS overhang (conventional siRNA); and a 20-base sense strand to generate a 0-nt PS overhang (blunt siRNA) (Figure 2A).

#### siRNA structure has no impact on compound tissue accumulation

To evaluate the effect of siRNA structure on tissue distribution, mice were injected via a single subcutaneous (SC) injection with one of the three different siRNA scaffolds (20 mg/kg). At 1-week post-injection, antisense strand accumulation was measured in tissues using PNA hybridization assay (37,38). In this study, we limited our focus on tissues where DCA-conjugated siRNA silencing was previously observed (13) and thus liver, kidney, spleen, lung, heart, muscle and fat were selected. For all siRNA scaffolds, two siRNA sequences—Huntingtin (*Htt*) (46) and Cyclophilin B (*Ppib*) (47)—were used to evaluate the relative impact of siRNA nucleobase composition on distribution profile.

We observed no significant difference in primary and secondary tissues accumulation profile between the two sequences (Figure 2B), indicating that nucleobase composition has no major impact on DCA-mediated tissue distribution. Contrary to unconjugated compounds (13), DCA-conjugated siRNAs were quantitatively retained (close to 100% of injected dose), and distributed to a wide range of tissues, with highest accumulation observed in liver. Surprisingly, the length of the PS overhang had no significant impact on DCA-mediated distribution across extrahepatic tissues (Figure 2B and C), except for fat, where blunt (0-nt overhang) siRNAs accumulated significantly more (2 fold, *P* < 0.05) than asymmetric (5-nt overhabg) and conventional siRNAs (2-nt overhang) compounds (Figure 2C). These findings suggest that tissue accumulation is primarily driven by the conjugate, with minimal contribution from siRNA sequence and structure.

#### siRNA structure significantly impacts efficacy

**Figure 3.**
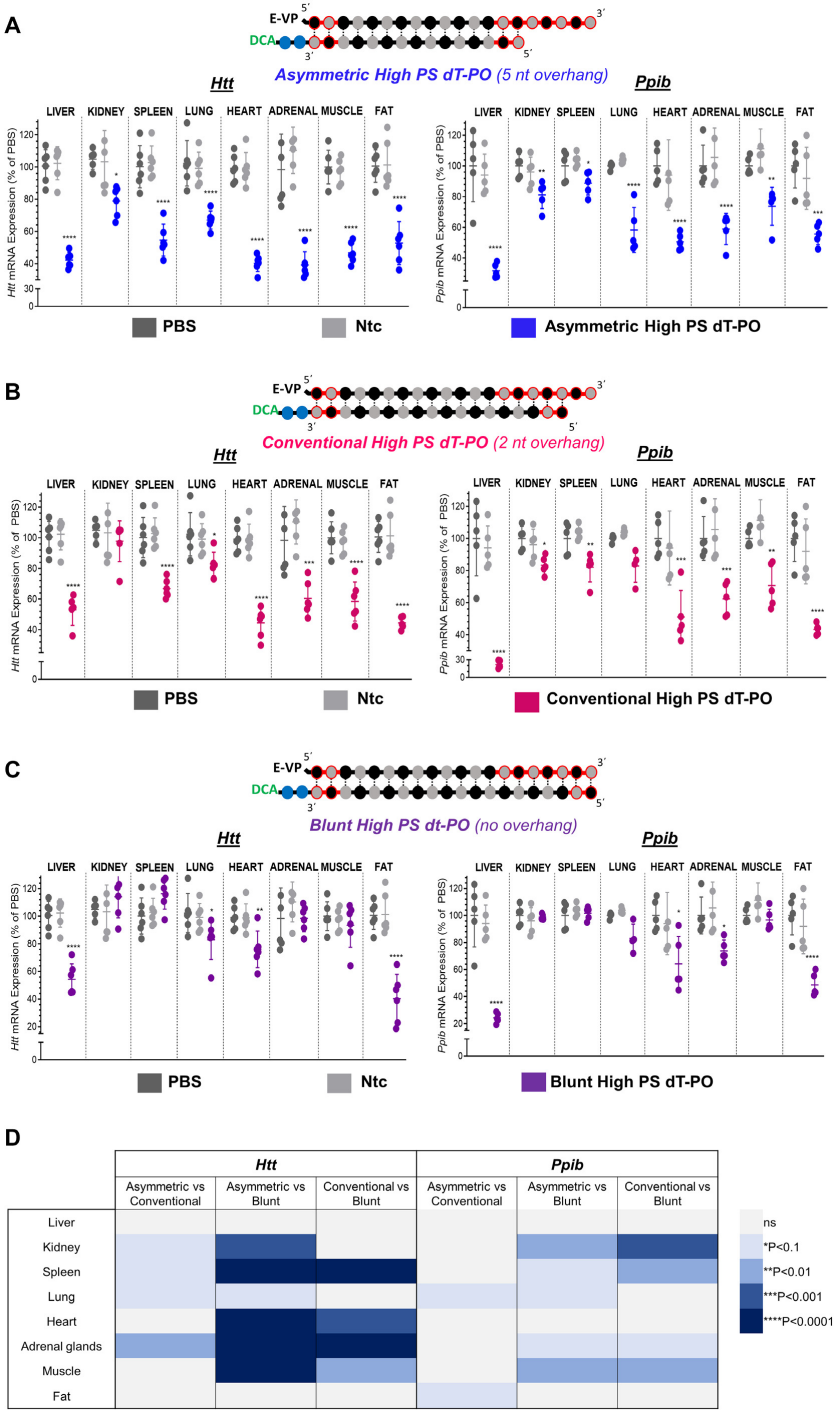
Presence of 5- or 2-nt PS-modified overhang enhances extrahepatic activity of DCA-conjugated siRNAs. Percent silencing in liver, kidney, spleen, lung, heart, adrenal glands, muscle and fat after subcutaneous injection of asymmetric (**A**), conventional (**B**) or blunt (**C**) DCA-conjugated siRNA targeting *Htt* (left panel) or *Ppib* (right panel) mRNA (*n* = 5–6 mice per group, 20 mg/kg). mRNA levels were measured using QuantiGene^®^ (Affymetrix), normalized to a housekeeping gene, *Hprt* (Hypoxanthine-guanine phosphoribosyl transferase) and presented as percent of PBS control (mean ± SD). Data analysis: One-way ANOVA with Dunnett test for multiple comparisons (****P< 0.0001, ***P< 0.001, **P< 0.01, *P< 0.1). (**D**) Heat map indicating the degree of statistically significant differences observed between silencing of asymmetric (5-nt overhang), convention (2-nt overhang) and blunt (0-nt overhang) siRNA scaffolds in various tissues after SC injection of DCA-conjugated siRNA targeting *Htt* or *Ppib* mRNA (*n* = 5–6 mice per group, 20 mg/kg). Data analysis: *t*-test. Presence of the overhang rather than length of it has a profound impact on observed enhancement in activity.

To evaluate if similar levels of tissue accumulation translates to similar silencing efficacy for each siRNA structure, mice were SC injected with a single dose (*n* = 5–6 per group, 20 mg/kg) of each DCA-conjugated siRNA variant targeting either *Htt* (46) or *Ppib* (47). Both targets have validated siRNA sequences available, and are expressed in a wide range of tissues at different levels (*Htt* low; *Ppib* high). Control mice were treated with either a non-targeting control (Ntc) siRNA or PBS. At 1-week post-injection, measurements of *Htt*, *Ppib* and *Hprt* (hypoxanthine-guanine phosphoribosyl transferase, a housekeeping gene) mRNA levels were performed in liver, kidney, spleen, lung, heart, adrenal glands, muscle and fat. Figure 3 shows the silencing efficacy of each compound in each tissue (compared to PBS, One-way ANOVA). We then determined whether there were statistically significant differences in silencing between the different scaffolds, as shown in the Figure 3D heatmap.

Ntc showed no significant reduction in target gene expression, indicating that the observed silencing is due to sequence-specific effects (Figure 3). Overall, functional efficacy of DCA-conjugated siRNAs was similar between *Htt* and *Ppib*. For both targets, DCA-conjugated siRNA enabled silencing up to 70% in liver and up to 50–60% in heart, adrenal glands, muscle and fat. However, the exact degree of silencing differed slightly between the two tested targets, likely due to differences in cell-type specific expression and the degree of nuclear retention of each mRNA.

**Figure 4.**
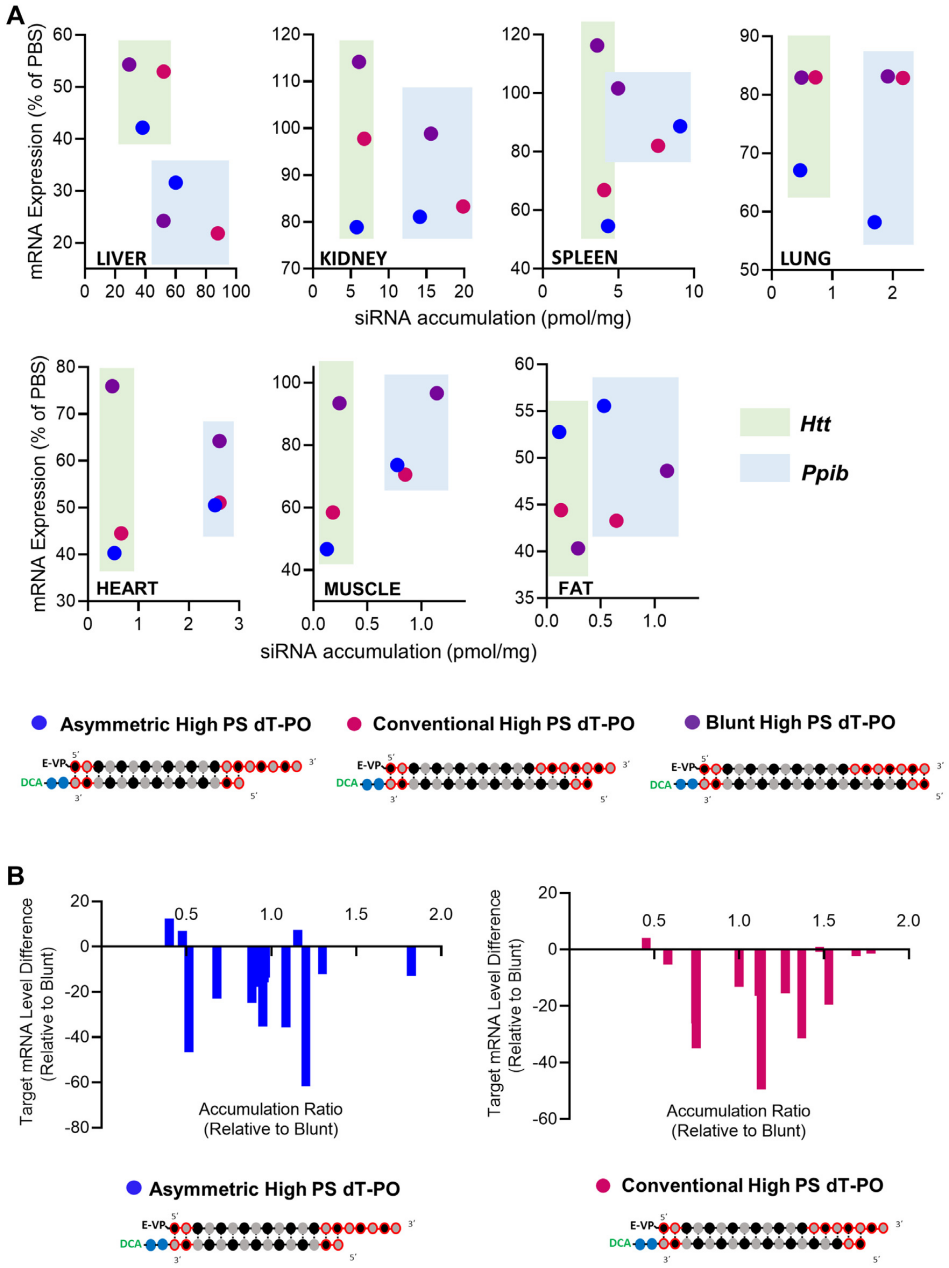
siRNA structure does not impact tissue accumulation but dramatically affects efficacy where asymmetric and conventional siNRAs induce the best silencing. (**A**) Graph correlating siRNA tissue distribution and efficacy in tissues for asymmetric, conventional and blunt siRNAs targeting *Htt* and *Ppib*. (**B**) Graph showing differences between mRNA level expression of asymmetric (left panel) or conventional (right panel) to blunt siRNAs for all tissues. All analyzed tissues and both gene targets are plotted in the same graph. Negative differences indicate a better induction of silencing with asymmetric or conventional siRNAs compared to blunt compounds.

All scaffolds showed significant silencing in liver (∼50–70%), likely due to the high accumulation in this tissue (Figure 3A–C). Surprisingly, despite the degree of extrahepatic accumulation being similar for all siRNA scaffolds (Figure 2B), we observed a significant impact of siRNA structure on extrahepatic gene silencing. For the blunt structure, silencing efficacy was highest in fat tissue (Figure 3C), correlating to its enhanced accumulation (Figure 2B). However, blunt siRNAs achieved statistically significant silencing in only two (out of six) other extrahepatic tissues (lung and heart when targeting *Htt*; heart and adrenal when targeting *Ppib*) (Figure 3C). Excluding fat, blunt siRNAs exhibited only 36% max silencing in extrahepatic tissues, much lower than that of asymmetric (61% max silencing) and conventional (55% max silencing) siRNAs (Figure 3A and B, respectively). Indeed, asymmetric siRNAs showed significantly better silencing (11–59% increases in silencing) compared to the blunt structure in six extrahepatic tissues when targeting *Htt* (kidney, spleen, lung, heart, adrenal glands and muscle), and five tissues when targeting *Ppib* (kidney, spleen, lung, adrenal and muscle) (Figure 4). Conventional siRNAs achieved greater silencing than blunt siRNAs (12–38% increases in silencing) in spleen, heart, adrenal glands and muscle (when targeting *Htt*), and in kidney, spleen, adrenal glands and muscle (when targeting *Ppib*, Figure 4).

Asymmetric siRNA exhibited the best extrahepatic efficacy overall, with silencing observed in all tissues (varying between 11 and 61%) (Figure 3A). Conventional siRNAs demonstrated slightly lower (but fairly comparable) activity, with silencing observed in seven out of eight tissues (varying between 17 and 55%) (Figure 3B). Asymmetric siRNA were slightly more potent compared to conventional siRNA in four of the extrahepatic tissues (when targeting *Htt*), and 2 extrahepatic tissues (when targeting *Ppib*, Figure 3D).

When correlating siRNA tissue accumulation and efficacy (Figure 4A), it is immediately clear that the silencing enhancement observed with asymmetric and conventional siRNAs versus blunt was not related to changes in accumulation. In general, Ppib targeting siRNAs accumulated slightly more in all tissues and for all structures than Htt targeting compounds, but the relative enhancement in efficacy stayed consistent between the two targets. Figure 4B shows change in silencing versus accumulation of asymmetric siRNA (left panel) and conventional siRNA (right panel) relative to blunt siRNA. All analyzed tissues and targets are plotted in the same graph. This visualization tool clearly shows (Figure 4B) an overall increase in activity for overhang-containing compounds in the majority of tissues (up to minus 50–60%).

Collectively, these results suggest that the presence (rather than length) of a PS overhang significantly enhances activity, possibly by influencing internalization mechanisms and/or degree of endosomal escape.

### PS content impacts DCA-siRNA distribution and efficacy

#### PS content affects siRNA distribution profile in the context of conventional siRNAs

For ASO, PS modifications define relative liver/kidney distribution. Fully PS ASOs preferentially distribute to liver due to tight serum protein binding. Decreasing the PS content on ASOs reduces serum binding affinity, shifting accumulation to kidney proximal epithelia, which retain a fraction of ASOs during clearance (21,48–49).

**Figure 5.**
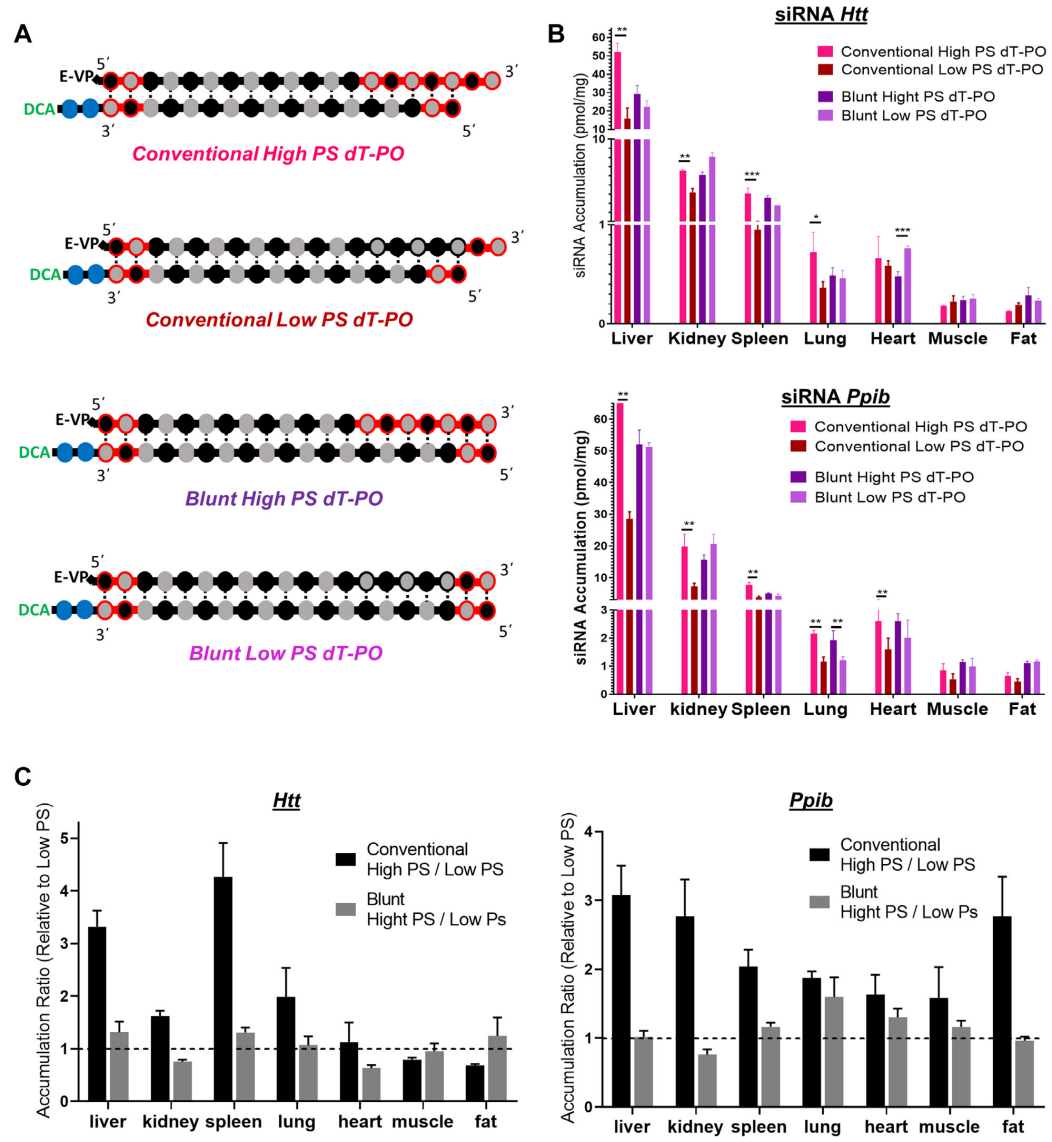
Increase in PS content enhances DCA-siRNA tissue accumulation in a context of overhang containing structures. (**A**) Schematic of siRNA scaffolds and PS content variation. (**B**) Bar graph showing accumulation of high-PS and low-PS DCA-conjugated siRNA targeting *Htt* (top) or *Ppib* (bottom) mRNA in liver, kidney, spleen, lung, heart, muscle and fat. siRNA accumulation measured 1-week after a single subcutaneous injection of DCA-siRNA (20 mg/kg, *n* = 5–6 mice per group ± SD) by PNA hybridization assay. Data analysis: *t*-test (***P< 0.001, **P< 0.01, *P< 0.1). (**C**) Bar graph showing the tissue accumulation ratio of High PS (13 PS) siRNA to Low PS (8 PS) siRNA targeting *Htt* (left) or *Ppib* (right) mRNA.

To determine whether the extent of PS modifications impacts extrahepatic distribution of siRNAs, we compared the distribution profile of DCA-conjugated siRNA with eight terminal PS modifications (two at each termini, ‘low PS’) versus 13 PS modifications (‘high PS’) in the context of conventional (2-nt overhang) and blunt (0-nt overhang) siRNA structures (Figure 5A). Levels of antisense strand accumulation were measured in liver, kidney, spleen, lung, heart, muscle and fat 1 week after SC injection of DCA-conjugated siRNA targeting either *Htt* or *Ppib* (Figure 5B).

In the context of blunt DCA-conjugated siRNA, change in PS content had minimal impact on tissue distribution profiles (Figure 5). However, for conventional siRNAs, the change in PS content significantly affected tissue accumulation. Specifically, low-PS compounds showed lower accumulation in most tissues—∼3- to 3.5-fold for liver, ∼1.5- to 3-fold for kidneys, ∼2- to 4-fold for spleen, ∼2-fold for lung, ∼0- to 2-fold for heart and muscle and ∼0- to 3-fold for fat (Figure 5C). These observations can be explained, in part, by the lower stability of ‘low PS’ overhang compared to the ‘high PS’ overhang in conventional siRNAs. Collectively, these results suggest that PS modifications likely promote siRNA accumulation and retention in tissues by preventing siRNA degradation and altering clearance kinetics.

#### High PS content has a negative impact on siRNA activity

**Figure 6.**
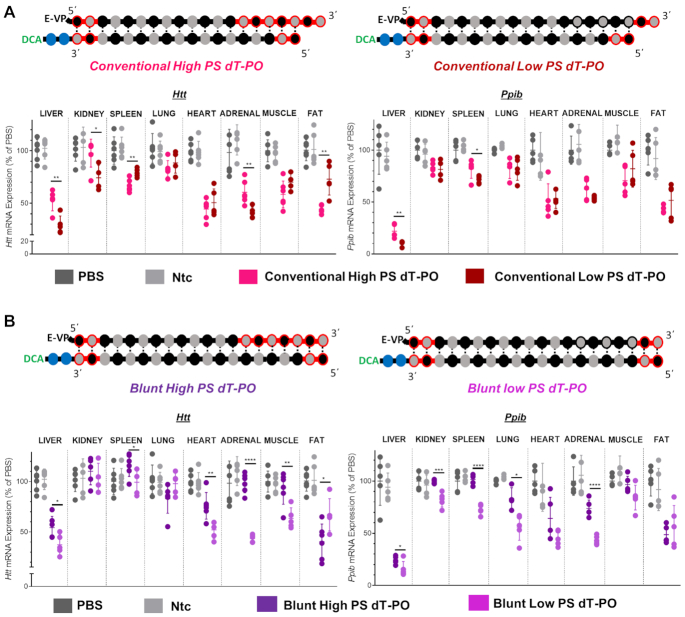
Increase in PS content negatively impacts siRNA functional efficacy in the majority of tissues. Percent silencing in liver, kidney, spleen, lung, heart, adrenal glands, muscle and fat after subcutaneous injection of high-PS and low-PS. (**A**) Conventional siRNAs or (**B**) blunt siRNAs targeting *Htt* (left panel) or *Ppib* (right panel) mRNA (*n* = 5–6 mice per group, 20 mg/kg). mRNA levels were measured using QuantiGene^®^ (Affymetrix), normalized to a housekeeping gene, *Hprt* (Hypoxanthine-guanine phosphoribosyl transferase) and presented as percent of PBS (phosphate buffered saline) control (mean ± SD). Data analysis: *t*-test (****P< 0.0001, ***P< 0.001, **P< 0.01, *P< 0.1).

To evaluate if accumulation correlates with silencing, target mRNA levels were measured in tissues after injection of low PS and high PS blunt and conventional siRNAs scaffolds targeting *Htt* and *Ppib*. The silencing efficiency of each compound in each tissue (compared to PBS, One-way ANOVA) is shown in Figure 6.

**Figure 7.**
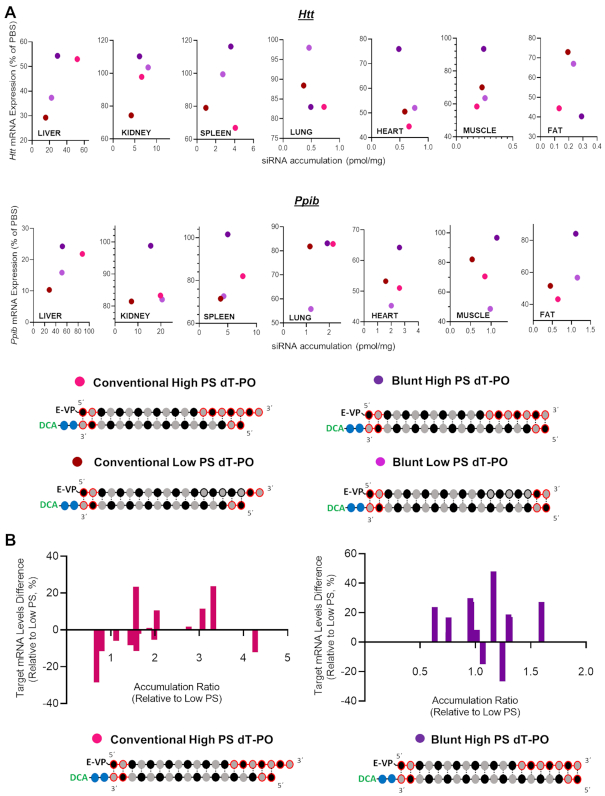
High PS content leads to a decrease of siRNA efficacy. (**A**) Graph correlating siRNA tissue distribution and efficacy in tissues for conventional high PS and low PS siRNAs and for blunt high PS and low PS siRNAs targeting *Htt* (upper panel) and *Ppib* (lower panel). (**B**) Graph showing differences between mRNA level expression of conventional high PS (left panel) or blunt high PS (right panel) to the corresponding low PS content variants. All analyzed tissues and both gene targets are plotted in the same graph. Positive differences indicate a better induction of silencing with low PS siRNAs compared to high PS compounds.

Suprisingly, despite of the enhancement in tissue accumulation observed with high PS conventional siRNAs, the increase in PS content have overall negative impact on activity (Figure 6). For conventional siRNAs, there were minor increases in silencing in fat and spleen (15 and 30%, respectively) with high PS compounds, but only observed for *Htt* and not *Ppib* targeting mRNA. At contrary, the clear negative impact of increase in PS context on efficacy and reversed correlation to accumulation can be observed in liver (Figure 6A). Despite an approximately 3-fold increase in accumulation with high PS compounds (52–65 pmol/mg for high PS siRNAs vs 14–29 pmol/mg for low PS siRNAs, Figure 5), the level of observed liver silencing was reduced from 73 to 47% for *Htt* (*P* < 0.01) and from 22 to 11% for *Ppib* (*P* < 0.01) (Figure 6A). When correlating siRNA tissue accumulation and efficacy (Figure 7A), it is stricking that for conventional siRNAs (pink and red dots, Figure 7A) even if high PS variants accumulated more in 12 tissues out of 14 compared to low PS siRNAs, high PS compounds induced statistically significant better silencing in only two tissues out of 14 (fat and spleen when targeting *Htt*) (Figures 6A and 7B). Collectively, these results suggest that an increase in PS content enhances conventional siRNA stability to increase tissue accumulation, but has a significant, negative impact on observed functional activity.

The negative impact of increased PS content on silencing was even more pronounced in the context of blunt siRNAs. Indeed, low PS blunt siRNA induced better silencing (17–30% increases in silencing) than their high PS counterparts for both targets in five out of eight tissues (Figure 6B). The effect was particularly pronounced in adrenal glands, where high PS siRNAs induced minimal to no silencing, and low PS compounds showed >55% reduction in both *Htt* and *Ppib* expression.The correlation between siRNA tissue accumulation and efficacy, clearly shows that for blunt structures (light and dark purple dots, Figure 7A), the number of PS did not significantly impact acumulation levels but affected activity where low PS siRNAs (light purple dots, Figure 7A) induced better silencing than high PS compounds (dark purple dots, Figure 7A). The differences between target mRNA levels of high PS siRNAs (Figure 7B, right panel) to low PS compounds (all tissues and targets plotted in the same graph) results in positive remaining mRNA expression (up to plus 50%) for the majority of tissues, indicating that low PS siRNAs were more potent than high PSvariants. These results suggest that, for blunt siRNAs with similar stability and distribution, a large number of PS modifications may alter protein binding inside the cell to impact siRNA trafficking, endosomal escape and the degree of functional silencing (27–29,50).

### The linker chemical composition has a strong impact on DCA-siRNA efficacy *in vivo*

A variety of linkers—e.g. triethyleneglycol (TEG) (15), disulfide (51) and carbon chain (13)—have been used for conjugated siRNAs and ASOs. Moreover, the introduction of a cleavable phosphodiester bond between the conjugate and the oligonucleotide has been shown to improve liver silencing of cholesterol-conjugated ASOs (36). However, there has not been a systematic evaluation of the impact of conjugated siRNA linker chemistry on extrahepatic activity and distribution *in vivo*.

**Figure 8.**
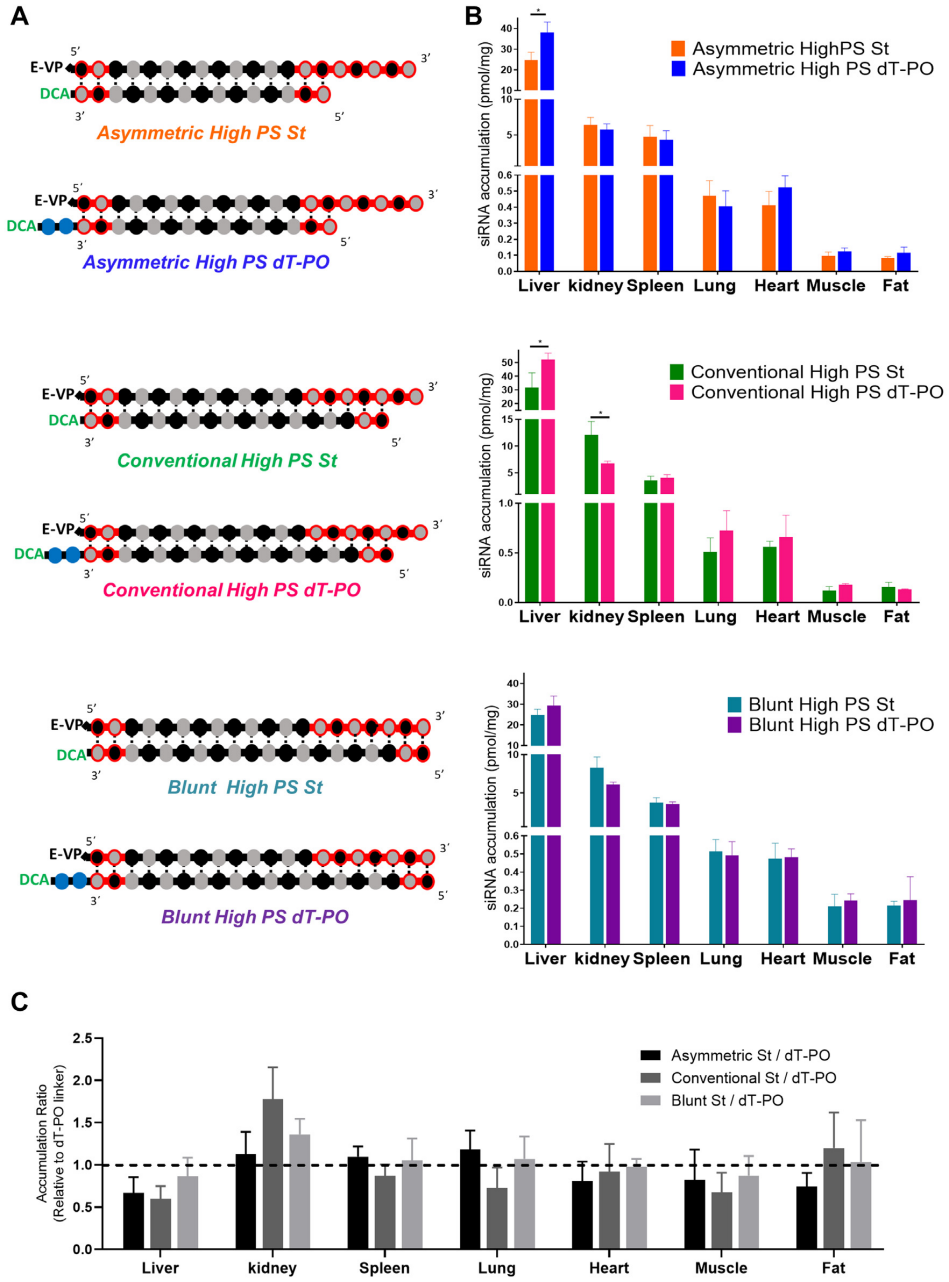
The presence of a cleavable linker between the conjugate and the siRNA does not impact siRNA tissue distribution and accumulation profile. (**A**) Schematic of siRNA chemical structures to evaluate the impact dT-PO versus stable carbon (St) linker on distribution. (**B**) Bar graph showing strand accumulation of asymmetric (upper), conventional (middle) or blunt (bottom) DCA-conjugated siRNA with dT-PO or stable carbon (St) linker in liver, kidney, spleen, lung, heart, muscle and fat. siRNA accumulation measured 1-week after a single subcutaneous injection of DCA-siRNA (20 mg/kg, *n* = 5–6 mice per group ± SD) by PNA hybridization assay. Data analysis: *t*-test (*P< 0.1). (**C**) Bar graph showing the tissue accumulation ratio of asymmetric, conventional and blunt dT-PO linked siRNAs to the corresponding variants St linked siRNAs.

In all experiments described thus far (Figures 1–7), siRNAs were connected to the DCA conjugate through two phosphodiester bonds between two thymidines (dT-PO). The phosphodiester DNA has limited *in vivo* stability, sufficient to support initial tissue distribution, but quickly degraded upon cellular uptake (52). To evaluate the impact of linker stability on DCA-siRNA tissue accumulation, asymmetric (5-nt overhang), conventional (2-nt overhang) and blunt (0-nt overhang) siRNAs were synthesized with either a cleavable dT-PO linker or a stable carbon (St) linker (Figure 8A).

**Figure 9.**
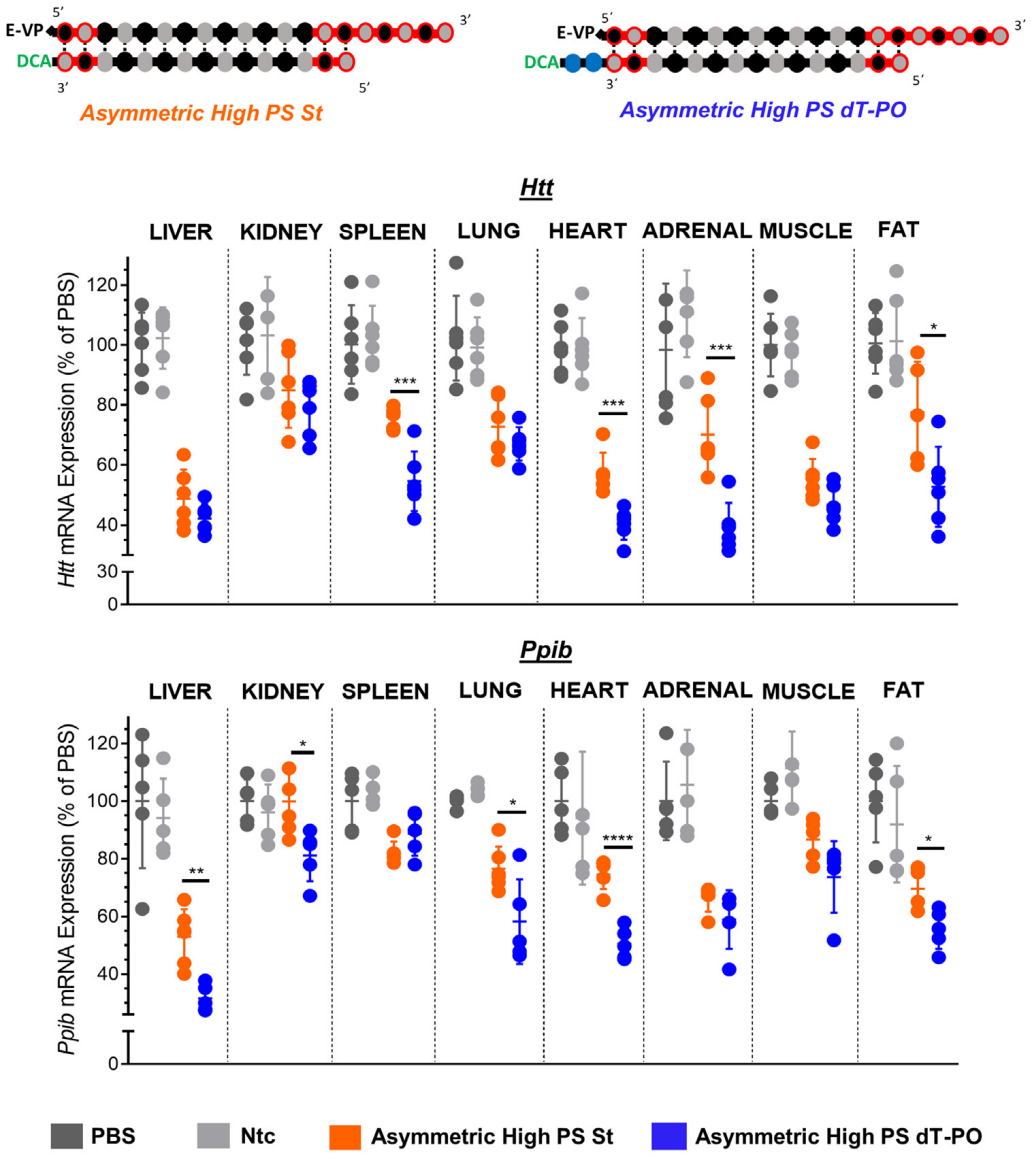
The presence of a cleavable linker enhances DCA-conjugated siRNA silencing in multiple tissues. Percent silencing of *Htt* (upper panel) and *Ppib* (bottom panel) in liver, kidney, spleen, lung, heart, adrenal glands, muscle and fat after subcutaneous injection of asymmetric DCA-conjugated siRNA with either dT-PO or stable carbon (St) linker into FVB/N mice (20 mg/kg, *n* = 6 per group). One-week post-injection, tissues were collected, and mRNA levels were measured using QuantiGene® (Affymetrix), normalized to a housekeeping gene, *Hprt* (Hypoxanthine-guanine phosphoribosyl transferase), and presented as percent of PBS control (mean ± SD). Data analysis: t test (****P< 0.0001, ***P< 0.001, **P< 0.01, *P< 0.1).

**Figure 10.**
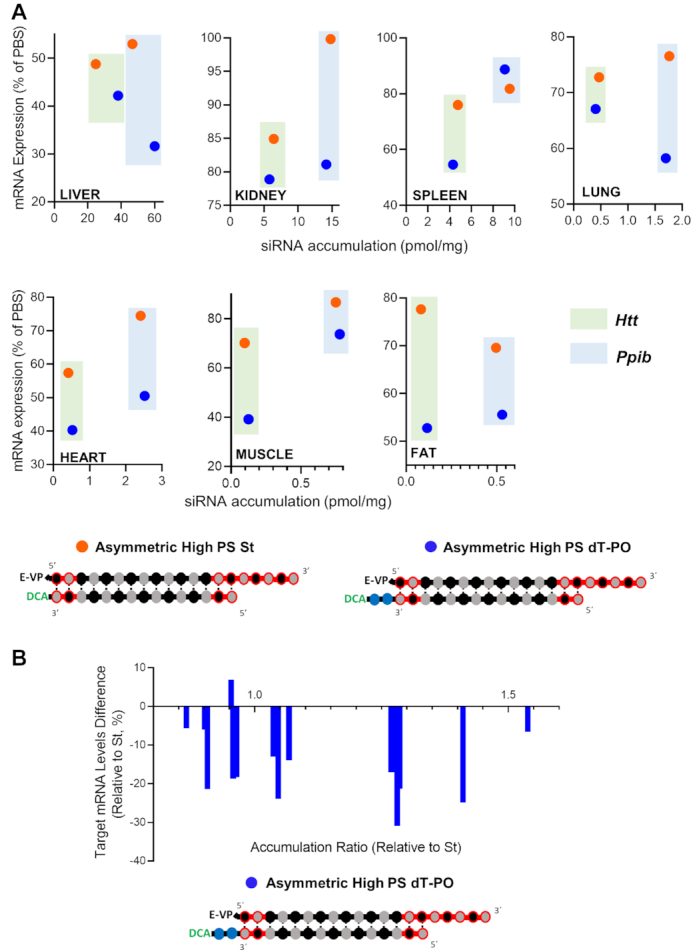
The linker chemistry does not impact tissue accumulation but dramatically affects efficacy where a cleavable linker induces the best silencing. (**A**) Graph correlating siRNA tissue distribution and efficacy in tissues for asymmetric siRNAs having a dT-PO linker and a stable carbon (St) linker targeting *Htt* and *Ppib*. (**B**) Graph showing differences between mRNA level expression of compounds having a dT-PO linker to siRNAs with a stable carbon (St) linker. All analyzed tissues and both gene targets are plotted in the same graph. Negative differences indicate a better induction of silencing with DCA dT-PO linked siRNAs compared to DCA stable carbon (St) linked compounds.

As expected, the nature of the linker had no significant impact on tissue distribution and accumulation profiles for any siRNA chemical structure (Figure 8B and C), indicating that dT-PO had sufficient serum stability to allow DCA-driven distribution. By contrast, the chemical composition of the linker did have a profound impact on tissue silencing levels (Figure 9). Specifically, the presence of a cleavable linker significantly improved *Htt* mRNA silencing in spleen (by 23%, *P* < 0.001), heart (by 14%, *P* < 0.001), adrenal glands (by 21%, *P* < 0.001) and fat (by 21%, *P* < 0.1); and significantly improved *Ppib* mRNA silencing in liver (by 24%, *P* < 0.01), kidney (by 20%, *P* < 0.1), lung (by 22%, *P* < 0.1), heart (by 25%, *P* < 0.0001) and fat (by 14%, *P* < 0.1) (Figure 9). The correlation between siRNA tissue accumulation and efficacy (Figure 10A) shows distinctly that the nature of the linker (dT-PO versus St) did not significantly impact siRNA tissue distribution (*x*-axis, Figure 10A) but had a dramatic effect on efficacy (*y*-axis, Figure 10A). DCA dT-PO linked siRNAs induced better silencing than DCA St linked compounds for both targets and in all tissues (except in spleen when targeting *Ppib* where silencing was similar with both compounds). The differences between target mRNA levels of DCA dT-PO siRNAs to DCA St compounds results in negative remaining mRNA expression (up to −30%) for the majority of tissues and both targets (Figure 10B), indicating that the presence of a dT-PO linker enhanced siRNA efficacy.

These results suggest that the use of a cleavable linker like dT-PO promotes silencing in tissues.

## DISCUSSION

Conjugation of oligonucleotides to a variety of chemical entities allows for modulation of bioavailability, tissues exposure and, in some cases, cell-type specific delivery (7–8,11,53). The recent approval of a fully chemically stabilized GalNAc siRNA, Givosiran, demonstrates the immense potential of conjugated siRNAs to treat genetic diseases (54). While a trivalent GalNAc allows specific delivery to hepatocytes (1–3,55), lipid conjugation enables functional delivery to a range of tissues beyond liver (13,14). Among lipophilic conjugates impacting oligonucleotide bioavailability, tissue distribution, kinetics of clearance and safety (10,13–14,37,56–57), we identified DCA as a conjugate that supports widespread extrahepatic distribution (13,14). However, DCA-siRNA accumulation and degree of silencing in extrahepatic tissues is less than what is generally observed for GalNAc conjugates in liver. Indeed, liver naturally accumulates drugs, including siRNAs, because it is a primary filtering tissue with high blood flow volumes and discontinued fenestrated epithelia (58), and thus represents a unique and highly favorable tissue for any drug targeting. To deliver compounds to other tissues, further optimization of conjugated siRNAs is needed. Here, we uncover the interplay between siRNA structure, chemical composition, and conjugate, and how it affects productive extrahepatic silencing. Such findings will pave the way toward using these classes of molecules for future therapeutic applications.

PS-modified oligonucleotides enhance protein binding and cellular uptake *in vitro* (28,30); and thus, are a primary factor defining oligonucleotide pharmacokinetics/dynamics (21,48,59). Yet, the impact of structural context (e.g. single- versus double-stranded; nature of conjugate) in which PS modifications are added can influence PS-induced protein interactions (60). In the context of a DCA conjugate, we found that the presence of a 5-nt or 2-nt PS overhang in asymmetric and conventional siRNAs (respectively) had no measurable effect on tissue accumulation profiles. This is likely because DCA, a highly hydrophobic moiety, binds serum protein so tightly (14) that the relative contribution of PS becomes less significant (9–10,13–14,57). Despite having no impact on overall tissue accumulation, the presence of the PS-modified single-stranded region did impact activity. Asymmetric siRNAs (5-nt overhang) induced statistically significant silencing in all tissues tested (16 out of 16), while blunt siRNA with an identical guide strand were active in only 50% of tissues. Structure variation (asymmetric versus blunt) had minimal impact on selected sequence activity *in vitro (**15**)*, but data presented here are limited to only two targets. Therefore, it is possible that other target sequences—i.e. those selected to optimally perform in a blunt structure—might generate different results. Furthermore, presence of the overhang could potentially enhance PAZ domain interactions and RISC loading (61–63); however, as the two chemical structures have very similar *in vitro* activity (15), this explanation is unlikely. Because the structures exhibit almost identical tissue accumulation, the observed functional differences are more likely due to the impact of the PS tail on intracellular localization (28), trafficking (29) and the degree of endosomal escape, which may be a rate limiting step for oligonucleotide activity (58). Interestingly, the length of the overhang had less of an impact on activity, with the 5-nt overhang inducing just slightly better silencing in ∼30% of tissues compared to 2-nt overhang siRNA. It is possible that the single-stranded PS region in both siRNAs is sufficient to mimic the behavior of ASOs; and thus, both alter trafficking and support efficient silencing (32–35). Collectively, there is a disconnect between level of accumulation and functional efficacy (previously observed with a range of different lipid conjugated siRNAs (13)), which indicates that both siRNA structural context and conjugate entity contribute to the intracellular behavior and cumulatively effect activity.

In addition to structural context, the extent of PS modifications on an oligonucleotide can influence their effect on stability (64), serum protein binding (21,31,48–49), cellular receptor binding (65), cellular trafficking (28) and nuclear localization (27–29). The resulting impact of PS content on distribution and efficacy of ASOs is well known, but the impact on extrahepatic distribution of conjugated siRNAs is less clear. Here, we found that decreasing PS content diminished tissue accumulation of asymmetric and conventional siRNAs—likely due to a decrease in stability of the single-stranded overhang. Accumulation of blunt compounds was unaffected because these compounds (no PS overhang) rely solely on the conjugate to define tissue accumulation. Surprisingly, an increase in PS content negatively impacted silencing for all structural contexts, but particularly for blunt compounds. It is possible that siRNAs with high-PS content bind too tightly or to a large variety of proteins inside the cells, which may alter trafficking, endosomal escape and reduce a fraction of compounds available for RISC loading (28–29,50). The exact mechanism requires further investigation, but our results suggest that unnecessary increases in PS content can be detrimental. Therefore, optimizing a specific balance between phosphodiester and phosphorothioate (PO/PS) content will be crucial for lipid-conjugated siRNAs to achieve maximum silencing in extrahepatic tissues. Indeed, optimization of PO/PS content for tricyclo-DNA ASOs (in Duchenne Muscular Dystrophy models) (66), and for oligonucleotides in the central nervous system has already been done, and is now a widely used strategy to achieve optimal efficacy, stability, safety (67). Another strategy for improving extrahepatic gene silencing would be to replace PS moieties with other chemical entities that stabilize siRNA, reduce cellular protein binding, and maintain the ability to be recognized by RISC and induce RNAi.

Until now, the relative contribution of linker chemistry on conjugated-siRNA *in vivo* efficacy was largely unknown, partially because linkers have often been considered an inert part of the chemical architecture. However, our findings demonstrate that, in the context of DCA-siRNAs, the use of a cleavable linker (dT-PO) had a profound impact on degree of silencing. We previously found that dT-PO is the simplest synthetic variant of a cleavable linker and, compared to mono-dT and rU linkers, has an optimal range of stability (unpublished data). Indeed, dT-PO (cleavable) and stable carbon (non-cleavable)-linker DCA siRNAs showed similar tissue accumulation profile confirming that dT-PO was stable enough in serum during the early stages of compound clearance. However, dT-PO significantly increased activity in several tissues, likely due to its cleavage inside the cells for enhanced siRNA endosomal escape. The positive effect of a cleavable linker on endosomal escape and efficacy has been observed for cholesterol-conjugated ASOs (∼35% increase in activity) (36), and for GalNAc-ASOs (Reversir) used to inhibit siRNA activity in liver (68). Although other clinical GalNAc compounds, such as Inclisiran, do not have a cleavable linker and are highly active, the use of a cleavable linker might be of higher functional significance in the context of lipid-conjugated siRNAs. For instance, after internalizion via endocytosis (30), lipid conjugates may be particularly susceptible to becoming membrane bound, limiting siRNA cytosol release. A cleavable linker may help overcome this issue. Overall, dT-PO is easy to synthesize, does not require special precursors, is non-toxic and is biodegradable; however, further work should more systematically evaluate diverse linker chemistries on siRNA *in vivo* activity.

Although our findings highlight the importance of optimizing chemical structure, there are many other modifications on siRNA that may influence extrahepatic efficacy. For instance, we used an alternating 2′-*O*-methyl and 2′-fluoro chemical modification pattern (first described by Allerson *et al.*) to enable conjugate-mediated siRNA delivery *in vivo* (18). While this modification pattern is highly efficient overall, Crooke *et al.* demonstrated that 2′-fluoro modifications within ASOs increase protein binding inside cells, leading to decreased silencing and increased ASO toxicity (69,70). Therefore, additional optimization of this modification pattern might profoundly impact siRNA potency and duration of effect *in vivo* (17); and thus, should be considered for enhancing extrahepatic silencing. Currently, further investigations are in progress to evaluate whether the optimized design of alternative 2′-*O*-methyl and 2′-fluoro compounds described here will be applicable to more OMe-rich siRNAs.

**Figure 11.**
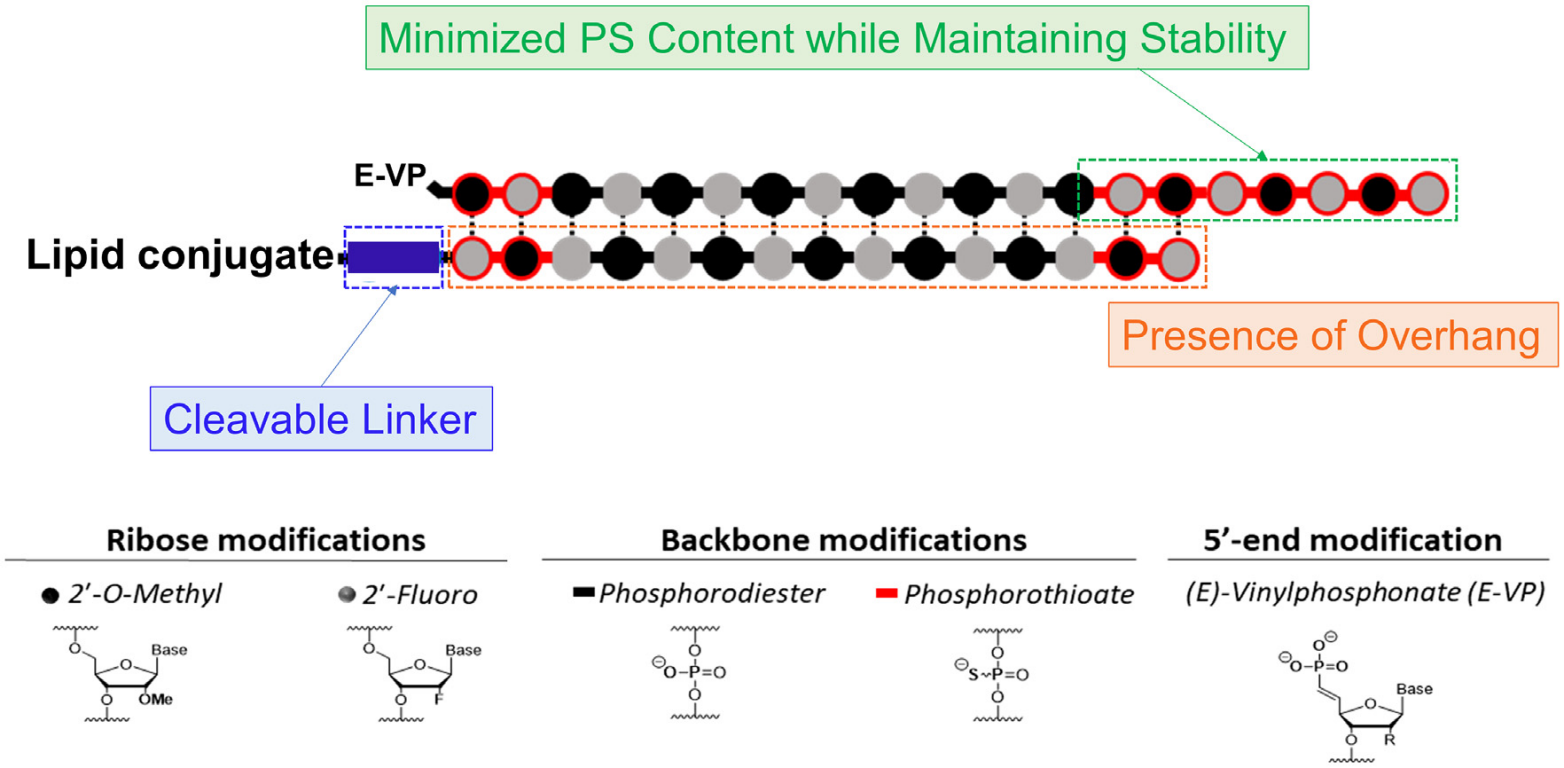
Optimized design of lipid conjugated siRNAs for enhancing extrahepatic silencing.

In summary, our data clearly demonstrate that the oligonucleotide chemical scaffold and architecture are principal factors to consider for optimizing the extrahepatic activity of siRNAs. Engineering strategies that alter structural asymmetry (e.g. 5- or 2-nt overhang versus blunt end) and linker chemistry (cleavable versus non-cleavable) can be used to fine-tune siRNA activity in kidney, spleen, heart, lung, muscle and adrenal gland without impacting tissue distribution. Moreover, engineering strategies that carefully balance PS versus PO content can be used to optimize siRNA stability without compromising functional efficacy in tissues. Overall, siRNA designs with an overhang, cleavable linker and minimal PS content (Figure 11) should support enhanced extrahepatic silencing. Our findings will guide the future design of conjugated siRNAs with optimal therapeutic profiles.
